# Information theory-based direct causality measure to assess cardiac fibrillation dynamics

**DOI:** 10.1098/rsif.2023.0443

**Published:** 2023-10-11

**Authors:** Xili Shi, Arunashis Sau, Xinyang Li, Kiran Patel, Nikesh Bajaj, Marta Varela, Huiyi Wu, Balvinder Handa, Ahran Arnold, Matthew Shun-Shin, Daniel Keene, James Howard, Zachary Whinnett, Nicholas Peters, Kim Christensen, Henrik Jeldtoft Jensen, Fu Siong Ng

**Affiliations:** ^1^ National Heart and Lung Institute, Imperial College London, London, UK; ^2^ Department of Physics, Imperial College London, London, UK; ^3^ Department of Mathematics, Imperial College London, London, UK; ^4^ Centre for Complexity Science, Imperial College London, London, UK; ^5^ Department of Cardiology, Imperial College Healthcare NHS Trust, London, UK; ^6^ Institute of Innovative Research, Tokyo Institute of Technology, Yokohama, Japan; ^7^ Department of Cardiology, Chelsea and Westminster NHS Foundation Trust, London, UK

**Keywords:** fibrillation, complexity, information theory, Granger causality

## Abstract

Understanding the mechanism sustaining cardiac fibrillation can facilitate the personalization of treatment. Granger causality analysis can be used to determine the existence of a hierarchical fibrillation mechanism that is more amenable to ablation treatment in cardiac time-series data. Conventional Granger causality based on linear predictability may fail if the assumption is not met or given sparsely sampled, high-dimensional data. More recently developed information theory-based causality measures could potentially provide a more accurate estimate of the nonlinear coupling. However, despite their successful application to linear and nonlinear physical systems, their use is not known in the clinical field. Partial mutual information from mixed embedding (PMIME) was implemented to identify the direct coupling of cardiac electrophysiology signals. We show that PMIME requires less data and is more robust to extrinsic confounding factors. The algorithms were then extended for efficient characterization of fibrillation organization and hierarchy using clinical high-dimensional data. We show that PMIME network measures correlate well with the spatio-temporal organization of fibrillation and demonstrated that hierarchical type of fibrillation and drivers could be identified in a subset of ventricular fibrillation patients, such that regions of high hierarchy are associated with high dominant frequency.

## 1. Introduction

Several mechanisms for sustaining myocardial fibrillation have been described [1]. However, the mechanism in each individual patient is less clear [2]. Understanding fibrillation mechanisms in specific patients would allow delivery of personalized care, which may improve patient outcomes. Ventricular fibrillation (VF) is a life-threatening arrhythmia and a common cause of sudden cardiac death. Catheter ablation for ventricular fibrillation (VF) is an emerging treatment option [3] that is guided by trigger and driver identification. Catheter ablation involves the delivery of energy (either heating from radiofrequency, or freezing) to create scar [4].

Atrial fibrillation (AF) is the most common cardiac arrhythmia in adults [5]. Catheter ablation is a commonly used procedure to aid maintenance of sinus (normal) rhythm. Pulmonary vein isolation (PVI) is the cornerstone of ablation for AF, and involves ablation encircling the pulmonary veins to electrically isolate the pulmonary veins, which harbour the triggers of AF, from the left atrium [6]. However ablation for persistent atrial fibrillation has limited single procedure success [7], and this may be due to the current one-size-fits-all approach. This limited efficacy has prompted a search for ablation strategies beyond PVI. These strategies usually involve delivery of additional ablation lesions (lesion sets) in specific areas of the atria. These generally aim to isolate areas responsible for initiation of fibrillation (trigger) or driver regions. A driver is a focal or localized source with fast, repetitive activity propagating outward from this source [8]. Many adjunctive lesion sets beyond PVI have been tested; however, none have demonstrated convincing efficacy [9].

Accurate identification of driver domains may be an important target in both AF and VF ablation. In AF ablation, if fibrillation is driven hierarchically by a spatially identifiable source, targeted ablation in that area or linear lesions to isolate that area may be effective in reducing AF recurrence [10]. Similarly, ablation of VF driver regions may reduce future VF episodes [3].

Originally derived from econometric time-series analysis [11], Granger causality (GC) analysis detects directed coupling between time series by considering the statistical dependency of a sink signal on the past of itself and another potential source signal. If the source signal significantly improves the predictability of the sink signal, then the source signal could be considered as ‘Granger-causing’ the sink signal. This type of analysis has initially seen broad applications in neuroscience [12]. Typical analysis consists of summarizing the statistical dependencies of neuronal data into functional connectivity networks for distinct activity or diseased brain states so that critical structures can be identified [13–15]. More recently, the analysis has been adopted in cardiac electrophysiology, where it has been applied to map dominant wavefront propagation patterns in fibrillation [16,17] and has been applied with vector analysis for driver identification [18,19]. Additionally, pairing with a traditional network analysis approach for treatment planning has also been proposed [20].

Interpretation of fibrillation propagation patterns from intracardiac electrograms (EGMs) has its unique challenges, as most signals are indirectly coupled and subjected to mixing of far-field activity. To identify the direct causality and also to eliminate common drivers resulting from far-field mixing, the multivariate extension conditional Granger causality (CGC) is required for EGM mapping applications. The multivariate analysis models the system as a vector auto-regressive (VAR) process, where the sink signal can be explained by a linear combination of the past of all variables within the system. In order to be considered as the direct Granger-cause, the past of the source signal then needs to significantly improve the fit of the model, after information from all other variables has been accounted for (i.e. conditioned). Direct application of CGC has been shown to yield less than optimal results in high-dimensional data [16,21], *a priori* assumptions of which signals could be physically coupled must be considered to eliminate implausible pairings, and specific signal pre-processing is required to satisfy the linear assumption [22]. Apart from enforcing plausible *a priori* assumptions, there are number of attempts to address the issues of estimation in high-dimensional data and linearity assumptions [12,13]. However, studies so far have focused on application in brain sciences and currently these developments have been missing in cardiac electrophysiology mapping.

Information theory offers a model-agnostic approach to estimating directed coupling, which has the potential of circumventing the limitations of the VAR-based GC [23]. In information theory, the signal could be considered as being worth a certain amount of information, quantified by Shannon’s definition of entropy. The entropy measures the amount of uncertainty given by the probability distribution of the signal, and has been applied as a mapping technique in cardiac electrophysiology [24]. In contrast to a linear predictability framework, nonlinear coupling of two signals could be quantified by considering the joint probability distribution of the two signals, which is given by the difference of uncertainty with and without observing the other signal. Direct nonlinear coupling removing the common coupling of a third variable can be discerned by replacing the distributions with conditional distributions conditioned on the common input variable. Under the conditional mutual information paradigm, transfer entropy (TE) was introduced to detect directed coupling in physical systems [25]. Multivariate extension of the measurement has been introduced in the form of partialized transfer entropy (PTE) [26], which also removes common sources, therefore measuring direct coupling effects. Both TE and PTE are sensitive to nonlinear coupling, but their performance still falls short when applied to systems of higher dimensions. To this end, dimension reduction was implemented by selecting only the most relevant variables to be conditioned [27–29], albeit at a much higher computational cost. These information theory based approaches have demonstrated advantages when applied to high-dimensional nonlinear systems [30]. Currently, only the non-directional measure, mutual information, has been applied for characterization of AF organization [31]. Additionally, there is no informative guide on how to apply information theory based causality measurement for fibrillation characterization or classification, especially when applied to high-dimensional data of advanced imaging modalities such as optical imaging or ECG imaging data.

In the present study, we aimed to apply recently developed information theory-based causality measures to describe myocardial fibrillation mechanisms in individual subjects. We consider the application of the direct directional measure of partial mutual information from mixed embedding (PMIME) to classify fibrillation dynamics. To understand the applicability of PMIME given limited and confounded data, we empirically assess the performance of PMIME against fully conditioned GC using simulated electrograms. To address the computational load issue, we extend the CGC and PMIME analysis to the subspace projections of high-dimensional synthetic data, and validate if fibrillation driver regions could be distinguished based on coupling network analysis. We then apply the analysis to experimental cardiac optical mapping data and clinical non-invasive ECG imaging (ECGI) data for organization characterization and classification of fibrillation dynamics.

## 2. Methods

### 2.1. Simulation

The synthetic signals were generated using the Fenton–Karma model in two dimensions [32,33]. Briefly, an explicit Euler scheme and a central difference scheme were used to solve the following system of PDEs:

2.1 $$\frac{\partial V}{\partial t}=\nabla \cdot (D\nabla V)+\frac{I_{{\mathrm{Na}}^{+}}(V,u)+I_{{\mathrm{Ca}}^{+}}(V,w)-I_{K^{+}}(V)}{C_{m}},$$

2.2 $$\frac{\partial u}{\partial t}=\frac{H(v_{c}-V)(1-u)}{\tau_{u}^{-}(V)}-\frac{H(V-v_{c})v}{\tau_{u}^{+}}$$

2.3 $$\mathrm{and}\;\;\frac{\partial w}{\partial t}=\frac{H(v_{c}-V)(1-w)}{\tau_{w}^{-}}-\frac{H(V-v_{c})w}{\tau_{w}^{+}},$$

where *V* is the dimensionless membrane potential and *u* and *w* are gating variables for sodium current $I_{{\mathrm{Na}}^{+}}$ and calcium current $I_{{\mathrm{Ca}}^{+}}$ respectively. *H* denotes the Heaviside step function and *D* is the diffusivity tensor. In all simulations, the tissue is assumed to be isotropic so that *D* was a diagonal matrix where diagonal elements were set to 0.001. The currents ($I_{{\mathrm{Na}}^{+}},I_{{\mathrm{Ca}}^{+}},I_{K^{+}}$) and the voltage-dependent time constant $\tau_{u}^{-}$ are updated according to

2.4 $$I_{{\mathrm{Na}}^{+}}(V,u)=\frac{u}{\tau_{d}}H(V-v_{c})(1-V)(V-v_{c}),$$

2.5 $$I_{{\mathrm{Ca}}^{+}}(V,w)=\frac{w}{2\tau_{Ca^{+}}}(1+\tanh\left(k(V-v_{c}^{Ca^{+}})\right)),$$

2.6 $$I_{K^{+}}(V)=\frac{V}{\tau_{0}}H(v_{c}-V)+\frac{1}{\tau_{r}}(V-v_{c})$$

2.7 $$\mathrm{and}\;\;\tau_{u}^{-}(V)=H(V-v_{u})\tau_{u1}^{-}+H(v_{u}-V)\tau_{u2}^{-}.$$

For all simulations, Neumman boundary conditions are assumed, and the schemes were iterated over a time step difference of 0.1 ms and a spatial difference of 0.25 μm. For experiments with high-dimensional data, the membrane potentials were used directly as the signal closely resembles typical optical and ECGI signals. For other simulations, uni-polar electrograms Φ were calculated by evaluating the following integral [34]:

2.8 $$\Phi (x_{c},y_{c})=\iint \frac{\nabla \;.\;D\nabla V}{\sqrt{{\left(x-x_{c}\right)}^{2}+{\left(y-y_{c}\right)}^{2}}}\;dx\;dy,$$

where (*x*_*c*_, *y*_*c*_) are Euclidean coordinates of the electrode. The electrograms are then filtered with a notch filter (10–200 Hz), squared, and the moving averages over a window length of 20 frames were used for subsequent analysis by either PMIME or CG [19]. Note that to avoid numerical problems, *x*_*c*_, *y*_*c*_ are not integers. Parameters used for simulation experiments can be found in table 1.

**Table 1.** Model parameters used for simulation. Simulations 1 and 2 were paced experiments. Simulation 3 was a stable vortex induced by cross-field pacing.

parameter	paced ECGs Simulations 1 and 2	stable vortex Simulation 3
*C*_*m*_	1	1
*v*_*c*_	0.13	0.13
$\tau_{u}^{+}$	3.33	10
$\tau_{w}^{-}$	11	65
$\tau_{w}^{+}$	667	1000
*τ*_*d*_	0.25	0.1149
$\tau_{{\mathrm{Ca}}^{+}}$	45	22
$v_{c}^{{\mathrm{Ca}}^{+}}$	0.85	0.85
*τ*_0_	8.3	12.5
*τ*_*r*_	50	25
*v*_*u*_	0.055	0.025
$\tau_{u1}^{-}$	1000	333
$\tau_{u2}^{-}$	19.2	40
*k*	10	10

### 2.2. Conditioned Granger causality index

For estimation of conditional Granger causality index, the convention of vector auto-regression via QR decomposition was adopted. For the multivariate conditional Granger causality conditioned on $Z_{1},Z_{2},\ldots Z_{M-2},$ variables. Estimating the conditioned Granger causality index of variable Y and variable X of *N* samples involves first fitting the data to the unrestricted and restricted models (asterisk) and are specified as follows:

2.9 $$X_{t}=\sum_{k=1}^{\;p}{\left[\beta_{k}\right]}_{X,X}X_{t-k}+\sum_{k=1}^{\;p}{\left[\beta_{k}\right]}_{Y,X}Y_{t-k}+\sum_{k=1}^{\;p}{\left[\beta_{k}\right]}_{Z_{1},X}Z_{1,t-k}+\cdots +\sum_{k=1}^{\;p}{\left[\beta_{k}\right]}_{Z_{M-2},X}Z_{M-2,t-k}+\varepsilon_{t}$$

and

2.10 $$X_{t}=\sum_{k=1}^{\;p}{\left[\beta_{k}^{*}\right]}_{X,X}X_{t-k}+\sum_{k=1}^{\;p}{\left[\beta_{k}^{*}\right]}_{Z_{1},X}Z_{1,t-k}+\cdots +\sum_{k=1}^{\;p}{\left[\beta_{k}^{*}\right]}_{Z_{M-2},X}Z_{M-2,t-k}+\varepsilon_{t}^{*},$$

where *p* is the model order, $\beta_{k}$ is the regression coefficient matrix for lag *k* ∈ {1, 2, 3, … , *p*} and ε is 1 × (*N* − *p*) vector which collects the Gaussian innovations. The conditioned Granger causality index can then be computed as the log-likelihood ratio of the restricted and unrestricted model, i.e.

2.11 $$CGCI_{Y\overset{X}{\to }}=\ln\left(\frac{\Sigma^{*}}{\Sigma }\right)=\ln(cov(\varepsilon^{*}))-\ln(cov(\varepsilon )).$$

To decide on a suitable model order *p*, to avoid underfit or overfit, the Akaike information criterion (AIC) was adopted to balance the complexity of the model and the fit of the model. Models of orders 1–15 were fitted, and the model with the lowest AIC was selected. After the selection of a model, the *F*-test was used to assess the significance of the Granger causal index, where the significance level *α* has been set at either 0.05 or 0.01. Other information criteria may be used in place of AIC; however, in our testing AIC appeared to be most robust among other common information criteria such as Bayesian information criterion (BIC) [35] and corrected Akaike information criterion (cAIC) [36] (electronic supplementary material, figures S4 and S5), while it is less conservative than other information criteria for choosing *p* (electronic supplementary material, figure S6).

### 2.3. Partial mutual information from mixed embedding

PMIME was used to detect linear and nonlinear direct coupling between signals. Notably, the algorithms progressively build an embedding vector from top correlated components in the sense of estimated mutual information with the future of the driven component. The final causal effect is only conditioned on and estimated from components in the embedding vector, thus avoiding issues with high dimension and ‘over-conditioning’ in the case of fully conditional GC [21]. For mutual information estimation, the *k* nearest neighbour method was used. For the parameter *k* of 5, 10, 15, the performance of PMIME is stable (electronic supplementary material, figure6) and the default (*k* = 5) was used.

Given the *M* components system, define $X_{t},Y_{t},Z_{1,t},\;\ldots ,Z_{M-2,t}$ to be the sets containing the respective lagged and original components, i.e. $X_{t}=\{X_{t},X_{t-1},\;\ldots ,X_{t-L}\}$, and let *W*_*t*_ denote the union of all sets, where *L* is the maximum time lag tested and is set to 15 for all analyses. The algorithm starts with an empty embedding vector $V_{t}^{0}$ where the superscript denotes the embedding cycle. At each embedding cycle, the embedding vector is then augmented by the component in *W*_*t*_ that maximally explains the future of the driven component $X_{t+1}$ by KNN mutual information (MI) estimates conditioned on previous embedding vector, i.e. for the *n*th embedding cycle the new component $W_{t}^{n}$ can be identified by

2.12 $$W_{t}^{n}=\arg\max_{W\in W_{t}}I(X_{t+1};W|V_{t}^{n-1}).$$

And the new embedding vector is

2.13 $$V_{t}^{n}=[V_{t}^{n-1},W_{t}^{n}].$$

The embedding scheme is repeated subject to a termination criterion:

2.14 $$\frac{I(X_{t+1};V_{t}^{n-1})}{I(X_{t+1};V_{t})}>A.$$

Here *A* is either a fixed constant set manually or an adaptive threshold based on the MI estimates statistic of surrogate data. For a set significance level *α*, *A* is the 1 − *α* quantile of surrogate mutual information estimates. Given the final embedding vector $V_{t}$, let $V_{i,t}$ denote the *i*th component of $V_{t}$, i.e. $V_{t}={\left\{V_{i,t}\right\}}_{i=1}^{n}$. The PMIME causal effects of *Y* to *X* is defined as

2.15 $$PMIME_{Y\to X}=\frac{I(X_{t+1};{\left\{V_{i,t}|V_{i,t}\in Y_{t}\right\}}_{i=1}^{n}|{\left\{V_{i,t}|V_{i,t}\notin Y_{t}\right\}}_{i=1}^{n})}{I(X_{t+1};{\left\{V_{i,t}\right\}}_{i=1}^{n})}.$$

An adaptive threshold based on temporal shuffled surrogate data was used for all experiments, except for the analysis of optical data, where a fixed threshold (0.95) was used to save computation time [27,29].

### 2.4. Benchmark definition

Let $A_{i,j}$ be the ground truth matrix where the *i*th row *j*th column element equals 1 if there is true direct directed coupling from *i*th signal to *j*th signal and anywhere else equals zero. And true direct directed coupling is defined as the *j*th signal recorded from the immediate next electrode from the one that records *i*th signal along the pacing direction. In other words, for uniform propagation, $A_{i,j}=1$ if *j* − *i* = 1 and $A_{i,j}=0$ for other cases. Let $A_{i,j}^{*}$ be the matrix containing the Granger causality or PMIME estimates with diagonal elements equal to zero, i.e. $A_{i,j}^{*}={\mathrm{GCI}}_{i\to j}$ or $A_{i,j}^{*}={\mathrm{PMIME}}_{i\to j}$. In benchmark tests, we did not consider the coupling strength given by either CGCI or PMIME; therefore the $A_{i,j}^{*}$ matrices are evaluated in the sense that all non-zero elements were mapped to ones. True positives (TP), false positives (FP) and false negatives (FN) are counted according to

2.16 $$\mathrm{TP}=\sum_{i}\sum_{j}A_{i,j}1_{x\neq 0}(A_{i,j}^{*}),$$

2.17 $$\mathrm{FP}=\sum_{i}\sum_{j}1_{x=0}(A_{i,j})1_{x\neq 0}(A_{i,j}^{*})$$

2.18 $$\mathrm{and}\;\;\;\mathrm{FN}=\sum_{i}\sum_{j}A_{i,j}1_{x=0}(A_{i,j}^{*}),$$

where $1_{x\neq 0}$ is an indicator function whose value is one ∀x≠0. After summation of the counts over all simulations the F1 score was used as benchmark:

2.19 $$F1=\frac{2\mathrm{TP}}{2\mathrm{TP}}+\mathrm{FP}+\mathrm{FN}.$$

### 2.5. Causality network analysis and surrogate testing

To quantify the amount of influence a single signal contributes to the global dynamic, we quantified degree contrast as the total causal effects caused subtracted total causal effects affected. Given the causal effects matrix $A^{*}$, the degree contrast (*C*_contrast_) of the *i*th signal is defined as

2.20 $$C_{\mathrm{contrast}}=\sum_{j}{\left[A^{*}-A^{*⊤}\right]}_{i,j}.$$

For application to high dimensional data, it was necessary to correct for multiple testing of the coupling measures. In our study to identify the dominant source and sink among potentially spurious causal effects, we followed a threshold procedure based on surrogate data statistics [13,37]. Surrogate data were generated from the original signals via phase randomization [38], which breaks the cross-correlation between signals while retaining the Fourier spectrum and moments statistics. A permutation of the surrogate data across subjects was used for the same analysis as the real data. This process was repeated for 500 permutations. From the *C*_contrast_ of phase randomized permuted data, we obtain an empirical cumulative distribution function (CDF) of the *C*_contrast_ estimates. Two thresholds can then be identified as the 2.5% and 97.5% quantiles of the CDF. For the real data estimates, we identify the signals below the lower threshold as sinks and the signals above the higher threshold as sources.

For spatial–temporal characterization, the connectance (*ρ*) of a network has m vertexes was defined as

2.21 ρ=∑i,j1x≠0(Ai,j∗)2×(m2).

For spectral clustering on a network, we used a heuristic algorithm that maximizes the modularity of the network [39].

### 2.6. Regions of interest and dimension reduction

High-dimensional data were projected to lower dimension linear subspace for analysis. The base region was removed from the whole heart shell and then the remaining vertexes were projected to a bullseye plot. Regions of interest (ROIs) were spatially defined based on the bullseye plot, the ventricular was split into 9 regions with the centre apex as a single region, and middle and basal sections each horizontally and vertically split into four regions. For the matrix containing the signals from a single ROI, the mean was subtracted from each signal followed by the singular value decomposition of the matrix. Provided the matrix is *M* by *T*, the first two components of the right singular matrix scaled by the corresponding eigenvalues were identified and used as representative time series for that ROI [13,40]. Causal effects were estimated for all representative time series, then summed for each ROI in order to calculate the inter-regional causality.

### 2.7. Rat ventricular fibrillation data collection

Our rat VF data collection protocol has been previously described [19]. Briefly, Sprague–Dawley rats were humanely killed and rapidly perfused *ex vivo* on a Langendorff apparatus. Programmed electrical stimulation was used to induce and sustain VF. Optical mapping was performed of the epicardial surface of the left ventricular anterior wall. The transmembrane voltage was recorded from optical mapping fluorescence data using our custom-made complementary metal-oxide semiconductor camera (Cairn Research, Faversham UK) using the potentiometric dye RH237 (25 μl of 1 mg ml^−1^ dimethyl sulfoxide; Thermo-Fisher, MA) and excitation-contraction uncoupler blebbistatin (10 μmol l^−1^; Tocris Bio-Sciences, Cambridge, UK) in 160×128 pixel resolution for a 10 s duration.

### 2.8. Clinical ventricular fibrillation electrocardiogram imaging data collection and ethics

Patients were recruited to undergo ECGI recordings during induced VF. Patients scheduled to undergo clinically indicated defibrillator threshold testing involving induction of VF were invited to wear a 252-electrode ECGI vest (Medtronic, USA) for the duration of their defibrillator implant procedure. Patients underwent low dose CT thorax to determine electrode positions and cardiac anatomy. Patients were recruited on basis of meeting clinical indications for cardiac resynchronization therapy defibrillator implantation for heart failure with left bundle branch block. VF was induced at the end of the implant procedure using either a shock-on-T or 50 Hz stimulation. The defibrillator sensed VF and delivered a shock. The shock was repeated at higher energy if the device failed to defibrillate initially and if this failed cutaneous defibrillator pads were on standby to defibrillate. All patients had successful sensing of VF and were successfully defibrillated using their implanted defibrillator lead. The study was approved by the local ethics board (13/LO/1440). The reconstructed epicardial electrograms were extracted from the ECGI system using custom made software and subsequently were analysed offline as described above.

## 3. Results

### 3.1. Evaluation of estimation performance with limited data availability

For multivariate analysis of *M* signals, the maximum dimension of the explanatory vector is *M* × *p*, where *p* is the maximum VAR modal order (CGC) or the maximum time lag (PMIME). Higher dimensions directly affect the model representation in (2.9) and (2.10) by additional terms, so more parameters need to be estimated and require longer data. This can be problematic for cardiac electrophysiology signals, as long duration time-invariant data can be difficult to obtain due to constraints on the collection of clinical data. Poor performance of CGC has been reported for ECG analysis [21]. A novel bottom-up approach for dimension reduction is an inherent part of the PMIME algorithm [29,41], which allows it to maintain high performance even when applied to data of high-dimensional neural mass systems [30].

**Figure 1.**
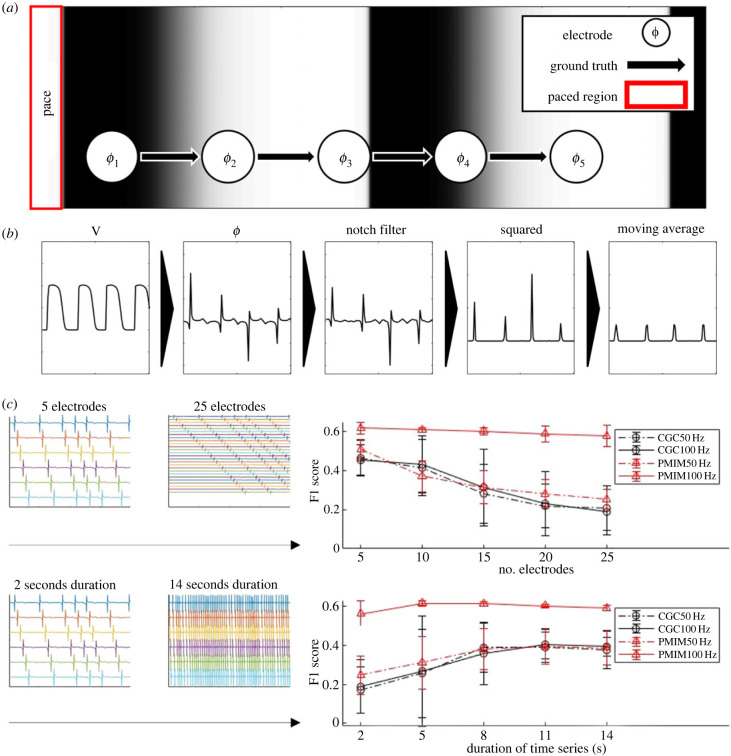
Sensitivity of CGC and PMIME to data availability. Performance evaluated by F1 scores of 100 simulations. (*a*) Schematic of the simulation protocol. (*b*) Signal processing steps for CGC and PMIME. (*c*) Performance results averaged over two critical values (*α* = 0.01, 0.05) and the range of the other factor; error bars correspond to the standard deviation. Top: effects of the number of electrodes included as inputs. Bottom: effects of time series duration.

In this section, we evaluate and compare the amount of data required for CGC and PMIME in the context of cardiac fibrillation. Periodic stimulation was delivered at the left end of the simulated tissue lattice, and electrodes were placed equally spaced along the wavefront propagation direction (figure 1*a*). We fixed the order and maximum lag to 15 and varied the number and duration of electrograms. F1 scores were calculated from pooled counts for each combination of number of electrodes and time series duration. The full results are shown in electronic supplementary material, figure S1. We tested the number of electrograms ranging from 5 to 25, where the duration of electrograms ranged from 2 s to 14 s with two significance levels (*α* = 0.05, 0.01) and two sampling frequencies (100 Hz, 50 Hz). Averaged results are shown in figure 1*b* to clarify the effect of a single factor. The performance of all algorithms monotonically decreases as the number of electrodes increases. Conversely, the performance increases as the duration of the time series increases. For both CGC and PMIME, across all parameter sets tested, a trade-off relationship can be observed between the number of electrograms and duration—accurate inference of coupling between a high number of electrograms is only possible with longer time series. For CGC, a sharper performance drop can be observed for conditions that fall outside the optimal range. CGC is outperformed by PMIME when data are limited or a large number of signals need consideration. The effects of the choice of critical threshold are minimal, while reduction in sampling frequency from 100 to 50 Hz negatively impacts PMIME but only affects CGC minimally. However, PMIME still outperforms CGC even at low sampling frequency.

### 3.2. Evaluation of estimation performance under Gaussian noise and far-field mixing

**Figure 2.**
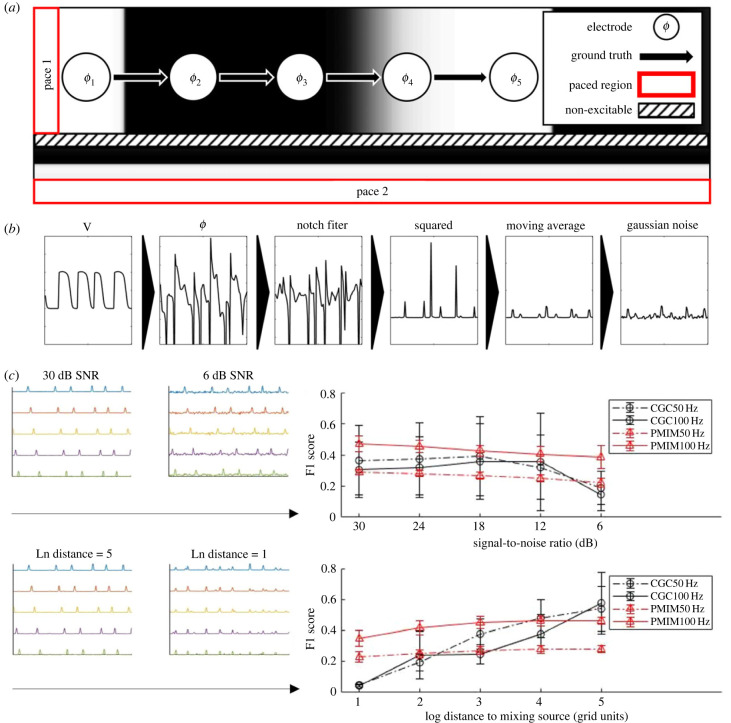
Sensitivity of CGC and PMIME to common confounding factors. Performance evaluated by F1 scores of 500 simulations. (*a*) Schematic of the simulation protocol. (*b*) Signal processing steps for CGC and PMIME. (*c*) Performance results averaged over two critical values (*α* = 0.01, 0.05) and the range of the other factor; error bars correspond to the standard deviation. Top: effects of Gaussian noise of varied signal-to-noise ratio. Bottom: effects of mixing of far-field activation at various distances.

To evaluate the performance of CGC and PMIME under potential confounding conditions, we generated electrograms that were corrupted by Gaussian noise from a tissue lattice with two independent sources of activation. As shown in figure 2*a*, electrodes were placed similarly to the previous set-up; however, a compartmentalized second domain was paced separately and the wavefront was orthogonal to the electrode alignment. Stronger far-field mixing effects were simulated by bringing the electrodes closer to the second domain. The number of electrograms was fixed to 10 and the duration is 8 s; signal-to-noise ratios (SNR) from 30 dB to 6 dB were tested. Far-field mixing strength was measured by the natural log-ed distance from the compartmentalization edge, the unit is in simulation units and ranges from 1 to 6.

Averaged results for individual confounding factors are shown in figure 2*b*. The performance of all algorithms decreased while PMIME was relatively more tolerant to profound mixing of a second source or Gaussian noise. Unexpectedly CGC increased slightly in performance under low Gaussian noise conditions before a sharp dropoff (50 Hz up to 18 dB, 100 Hz up to 12 dB). This has also been reported in a similar task [21]. While PMIME at 100 Hz still outperforms CGC at most settings, the performance of PMIME dropped significantly when the sampling frequency was decreased to 50 Hz. Full results calculated from 500 experiments are shown in electronic supplementary material, figure S2. Notably, lowering the critical threshold positively affects PMIME under confounding factors where it previously had no effects on the performance of PMIME in unconfounded conditions.

### 3.3. Validation of analysis applied to subspace projections of high-dimensional data

Reliable characterization of fibrillation dynamics in optical imaging or ECG imaging data requires accurate delineation of the hierarchy among thousands of signals. PMIME implements a forward selection scheme and is applicable to high-dimensional data; however, the computation time to iterate through the complete set of variables imposes challenges for application, where often a large number of surrogates must be analysed as the negative control for the signal processing pipeline [22,37]. Singular value decomposition (SVD) can be applied to a small number of regions of interest; the analysis is then applied to data in a lower dimensional subspace spanned by the top few eigenvectors of the decomposition [13,42]. Intuitively, this summarizes the dynamic in a few activation patterns that are often termed modes or principal components. The time-series analysis can then be applied to trajectories that correspond to each activation pattern. In the context of cardiac electrophysiology, this has been applied with the bi-variate GC to show rotor dynamics driving fibrillation in optically mapped tissue cultures [40]. However, spurious reciprocal causality is also reported. We extend this to multivariate analysis CGC and PMIME and validate their use for identifying fibrillation drivers in simulation experiments.

**Figure 3.**
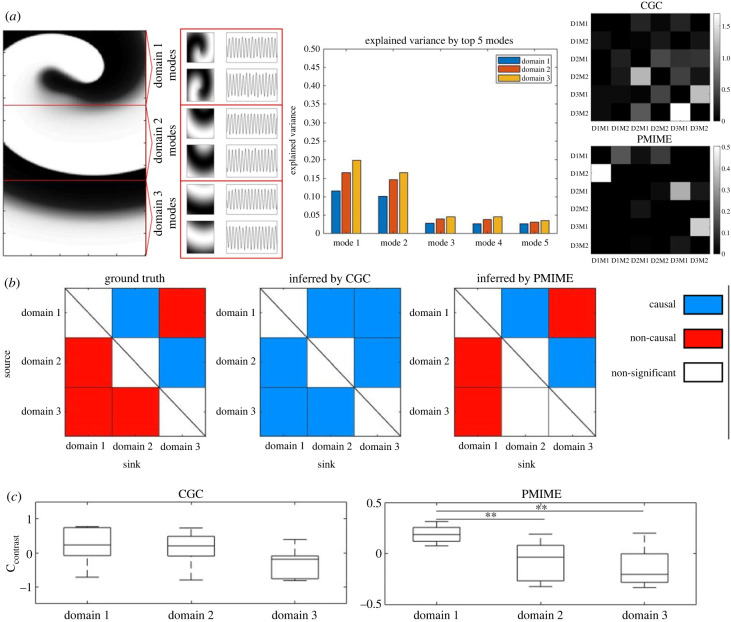
Comparison of CGC and PMIME when applied to data projected to linear subspace. (*a*) Simulation set-up and example of singular value decomposition (SVD) of the high-dimensional data followed by causality analysis. (*b*) Comparison of CGC and PMIME in determining the causal direction in high-dimensional data. Causal or non-causal by each measurements are based on Mann–Whitney *U*-test of 500 surrogates versus 10 simulation data. (*c*) Comparison of differentiating driver versus non-driver region based on *C*_contrast_ of CGC and PMIME causal networks. Asterisk indicates significance of Tukey’s HSD test: *p < 0.05; **p < 0.01.

Experiment set-up and SVD-based analysis are depicted in figure 3*a*. As shown in the left section, the vortex dynamic is induced at the top of a 500 × 200 rectangle tissue lattice. The first second of the simulation has been cut off to exclude the initialization and crossfield pacing phase. The resulting data for analysis had a total length of 8 s. The data are then split into three equal-sized domains while the rotational driver is always located in the top domain (domain 1). SVD is then applied to each domain and the top 2 modes are selected for analysis. The number of selected modes was set to 2 based on their importance measured in the total variance explained by them. The explained variance of the top 5 modes for each domain is shown in the histogram, which clearly identifies that the first two modes are the only modes that explained more than 5% of the data variance. Multivariate analysis is then applied to the selected mode trajectories and obtains the causal effect matrix of all modes. The causal effects are then combined for modes of the same domain to obtain the inter-regional measurement.

Inter-regional causal effects of 10 experiments and 100 surrogate data are summarized in figure 3*b*, and complete results are in electronic supplementary material, figure S3. To identify the causal region pairing, we follow the convention of surrogate data testing for both CGC and PMIME. The surrogate data in this experiment were generated using spatial and subject-wise permutations of the real data [13]. The surrogate data serve as the negative control for the spurious causal effect here. Notably, causal effects estimated by CGC of real data are statistically significantly higher than surrogates for all possible pairs (*p* < 0.01, Mann–Whitney *U*-test). By contrast, for causal effects estimated by PMIME, real estimates are higher than the surrogate for direct pairs along the propagation direction (positive *Z*-score for 1 → 2 and 2 → 3, *p* = 0.017 and *p* < 0.01, respectively, Mann–Whitney *U*-test). For all other pairs, PMIME real estimates are lower than the surrogate or non-significant. PMIME can eliminate indirect cause (negative *Z*-score for 1 → 3 in electronic supplementary material, figure S3; *p* < 0.01, Mann–Whitney *U*-test) and is immune to spurious results for most reciprocal pairs (negative Z score for 2 → 1, 3 → 1 but 3 → 2 in electronic supplementary material, figure S3; *p* < 0.01, *p* < 0.01 and *p* = 0.56, respectively; Mann–Whitney *U*-test).

We then examined if the hierarchy of the three domains could be distinguished based on the network measure of the causal networks. Degree contrast (*C*_contrast_) was calculated for each domain from the causal network of either CGC or PMIME and employed as a hierarchy measure (2.20). The results for the 10 experiments are summarized in figure 3*c*. *C*_contrast_ estimated from CGC networks showed a trend where the measurement is higher for the driver domain (domain 1) and lower for domains that are further away from the driver. However, the results were not statistically significantly different between any domains (one-way ANOVA). *C*_contrast_ derived from PMIME networks also showed the same trend with larger margins between the domains. The driver domain had a significantly higher *C*_contrast_ when compared to the other two domains (domain 1 versus 2 and 1 versus 3, *p* = 0.047 and *p* < 0.01, respectively, one-way ANOVA followed by Tukey’s HSD).

### 3.4. Spatial–temporal characterization by PMIME network measures

**Figure 4.**
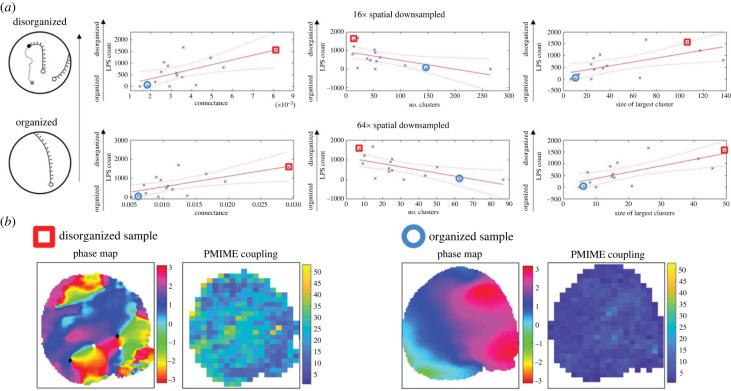
Applying network analysis to PMIME network for spatial–temporal characterization of fibrillation dynamics. (*a*) Network measures regressed to the phase mapping measure, the number of locations occupied by phase singularities (LPS). The columns, left to right, show connectance, number of clusters and size of the largest cluster, where the first and second row are 16× and 64× spatial down-sampled data, respectively. (*b*) Example phase map and node degree of bidirectional PMIME network. Extremities of the organization spectrum are shown, where the respective data points in the regression are marked by red box: disorganized and blue circle: organized. Asterisks denote the significance of Pearson’s correlation tests, *n* = 15: *p < 0.05; **p < 0.01.

Network measures of causal effects networks or correlation networks have been applied for spatial–temporal characterization of fibrillation organization [17,19,31]. To examine if basic network measures of PMIME network can characterize the spatio-temporal organization of fibrillation dynamics given spatially down-sampled data, we made use of high-resolution optical mapping data of ventricular fibrillation, where phase mapping was employed as the ground truth measure of organization. The number of locations occupied by phase singularities (LPS) were tracked historical locations of rotational activities or breakups of wavefronts, and is a measure of the degree of disorganization of fibrillation [19,43]. By correlating the phase mapping measurement in high-resolution optical imaging rat VF data (figure 4*a*), we examined network measures in two spatial down-sample settings (16×, 64×). PMIME network measures correlated well with the fibrillation organization spectrum, such that nodes within network of disorganized heart are associated with higher number of bidirectional couplings, owing to changing wavefronts through the time course of imaging. This is shown by regression analysis of network theoretical measures in figure 4*a*. The columns are network measures regressed on LPS and rows corresponded to the down-sample settings (top: 16×; bottom: 64×). Connectance describes how well the nodes are connected by the network and is found to be positively correlated to LPS in either setting. Next, we examined the network topology via spectral clustering. We found the number of clusters is negatively correlated with LPS whereas the size of the largest cluster is positively correlated with LPS. Such results are consistent for different spatial sampling settings. Hearts at the extremities of the organization spectra are shown in figure 4*b*, where the phase map and node degree (number of bidirectional couplings) highlight the association of phase measure and network measure. The network measure shown was estimated from 16× down-sampled data.

### 3.5. Hierarchy characterization of clinical fibrillation data

**Figure 5.**
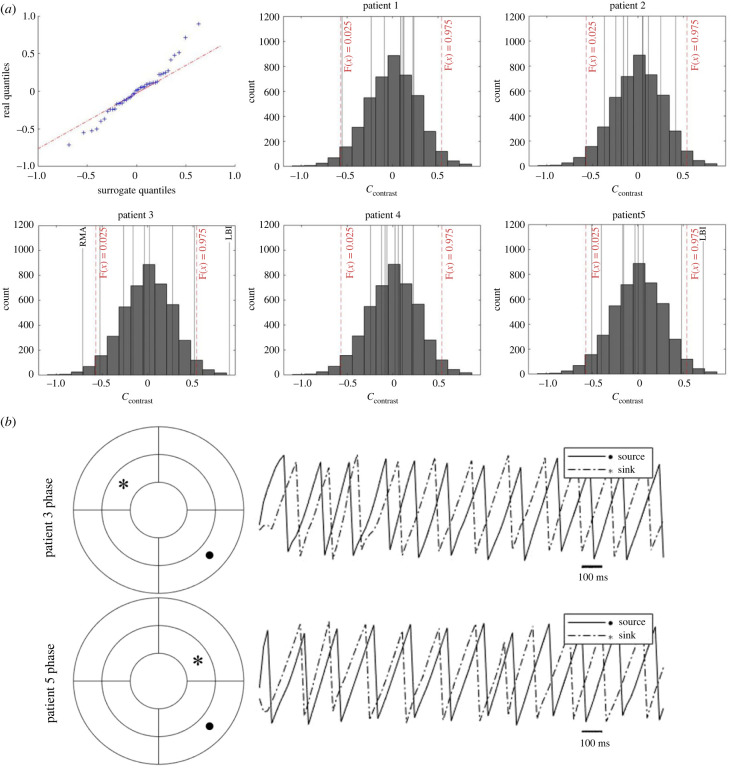
Applying network analysis for identifying driver region in clinical VF data. (*a*) Quantile–quantile plot of real (*n* = 5) versus surrogates (*n* = 500) *C*_contrast_ distribution, and real regional *C*_contrast_ for each patient (black vertical line) compared to surrogate *C*_contrast_ distribution (grey box). The upper and lower bound of 2.5–97.5th percentile range of surrogate distribution is indicated by red dashed lines. Regions outside the range are labelled. (*b*) Example signal phase traces from the source and sink regions identified.

As a proof-of-concept for identifying hierarchical fibrillation dynamics, we applied our analysis to a unique set of human ventricular fibrillation data acquired via non-invasive ECG imaging. Currently, the study of human ventricular fibrillation is limited due to the scarcity of data. Clinically observed VF is unlikely explained by a single mechanism, as either organized or disorganized dynamics have been reported [1,3], and spatially confined drivers could be identified subjectively. We demonstrate that it is possible to identify drivers objectively by comparing the theoretical measure *C*_contrast_ of patient data versus phase randomized surrogates. The ventricular shell was divided into 9 regions according to the projections to the bullseye plot. The region names and their acronyms are in table 2. Regional *C*_contrast_ was calculated based on the unique causality network of each patient or each permutation of surrogate. In total, 500 phase randomized surrogates were generated as the negative control for spurious causality, and the distribution of surrogates *C*_contrast_ were compared against the real data in figure 5*a*. The *C*_contrast_ distribution difference in real and surrogate data is shown by the quantile–quantile plot. The real data *C*_contrast_ distribution are skewed to the tails, suggesting overall the VF dynamics are more hierarchical than random data. The distribution of regional *C*_contrast_ for each individual patient was compared against surrogate distribution. It can be found that for patients 3 and 5, *C*_contrast_ of three regions (1 for patient 5, 2 for patient 3) falls clearly outside of the 2.5–97.5th inter-percentile range of the surrogate distribution, and these regions can be identified as a fibrillation driver region or sink, depending on whether they were above or below the range. Based on this definition, the LBI region was the fibrillation driver in both patients. The RMA is a sink for patient 3, whereas patient 5 did not have a dominant sink below the threshold. By contrast, regional *C*_contrast_ for patients 1, 2 and 4 all lie within the surrogate inter-percentile range and a hierarchical organization could not be clearly defined. Figure 5*b* shows example phase traces from the source and sink regions of the hierarchical cases. Note that the LMA trace was included as it is the region with the lowest *C*_contrast_ for that patient. The traces are of the beginning 2 s of VF, where it can be seen that the source trace maintains the lead of the phase cycle.

**Table 2.** Ventricle region acronyms and their full forms.

acronym	full form
AP	apex
RMA	right mid anterior
LMA	left mid anterior
LMI	left mid inferior
RMI	right mid inferior
RBA	right basal anterior
LBA	left basal anterior
LBI	left basal inferior
RBI	right basal inferior

**Figure 6.**
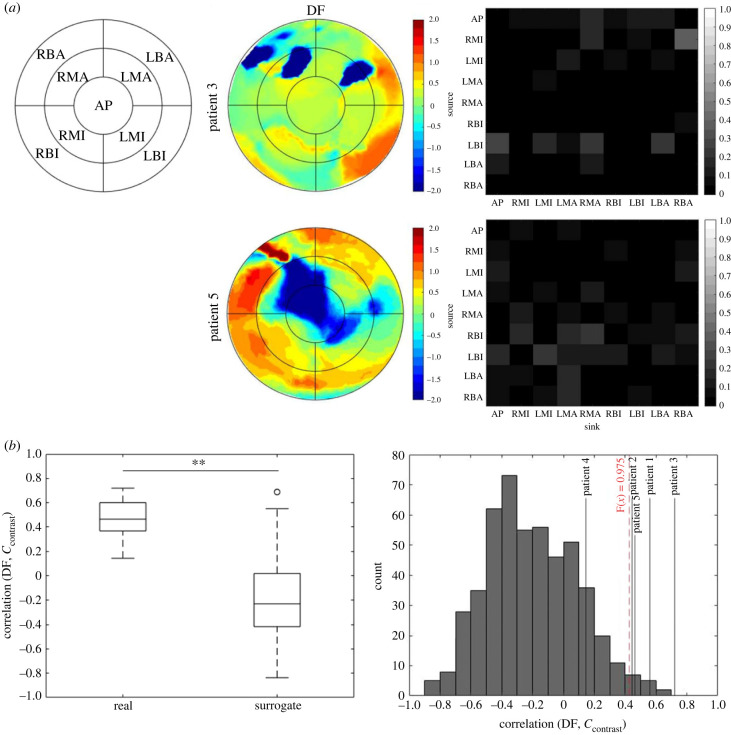
Correlation of the theoretical measure *C*_contrast_ with empirical driver marker dominant frequency (DF). (*a*) *Z*-scored DF heat maps and corresponding PMIME network adjacency matrix of the hierarchical cases. (*b*) Comparing region-wise correlation coefficients of DF and *C*_contrast_ of real and surrogate data. Box-plots are the coefficient of real (*n* = 5) and surrogates (*n* = 500), where each *n* is one patient or a permutation of nine regions. Right-hand panel shows the correlation in each patient (black lines) compared to the surrogate correlation distribution (grey box). The red dashed line indicates the upper 2.5% cutoff of surrogate distribution. **p < 0.01.

To shed light on the potential driving mechanism of VF, we correlated the hierarchical measure *C*_contrast_ with dominant frequency (DF), which is often regarded as an empirical marker for a fibrillation driver region [44,45]. The DF maps and the corresponding PMIME network adjacency matrix for the hierarchical cases are shown in figure 6*a*. DF has been *z*-scored to highlight the gradient within the patient. In either case, the main causal direction seems to be from high DF regions to low DF regions. Figure 6*b* shows the correlation coefficients of *C*_contrast_ versus DF for each patient and the 500 surrogates. The DF and *C*_contrast_ were moderately positively correlated in real data, which is statistically significantly higher than surrogate (*p* < 0.01, two-sample *t*-test). Examining each patient individually, the correlation coefficient of all but patient 4 was above the 2.5–97.5th inter-percentile range of the surrogate. Our results suggest for most cases high DF regions may indeed have a driving role in sustaining VF; however, the non-hierarchical case could not be ruled out and requires further study with a larger dataset.

## 4. Discussion

In this study, we describe the novel application of PMIME to detect direct directional nonlinear coupling of cardiac electrophysiology signals, specifically during cardiac fibrillation. Clinical recordings of cardiac fibrillation often have several challenges, including high dimensionality, short duration and far-field activity. We show that PMIME has several advantages over fully conditioned GC in analysing fibrillation dynamics. PMIME performs better when given short and high-dimensional data and the performance of PMIME is robust to mixing of Gaussian noise and far-field activity. We extended the analysis to subspace projections of high-dimensional data, and validated that PMIME, but not CGC, network correctly captures the hierarchy between different regions of a cardiac chamber in fibrillation. We found that PMIME network measures correlate well with standard organization measures and are consistent even when applied to spatially down-sampled data. By testing the network measures against phase randomized surrogates, we demonstrate that fibrillation drivers could be identified in clinical VF in our analysis. The analysis also provides supporting evidence that early VF could be driven by high DF sources.

High-dimensional data, such as intracardiac EGM recordings with multipolar catheters, in general require longer recordings in order to estimate the large number of parameters. Given the time constraints of a clinical procedure, long recordings in multiple areas of the chamber of interest (left atrium for AF, ventricles for VF) are not feasible. PMIME is therefore theoretically an ideal analysis technique to overcome the limitations of clinical data. In our analysis, PMIME was vulnerable to low sampling frequency; however, this is not an issue clinically, as sampling frequencies of 1000 Hz or more are common in clinical recording systems. Additionally, PMIME is able to identify direct causality and eliminate indirect causality with higher accuracy than CGC. In our testing in simulated electrogram data, CGC performed comparable to testing performed by other groups [21]. Under the same setting, PMIME vastly outperformed CGC given adequate sampling frequency. The difference between the two algorithms could not be explained by the data requirement for high-dimensional data alone. As we have shown, even when implemented with dimensionality reduction, the CGC still detects spurious reciprocal causal pairs, similar to those reported by Biton *et al.* [40]. In our *in silico* validation, the reciprocal causal pairs from non-rotational domains to rotational domains are either non-significant or rejected by PMIME. This appears to be due to PMIME being sensitive to the nonlinearity of the system where CGC fails and reaffirms the advantage of PMIME when applied to signals of nonlinear systems [30]. Our study has assessed PMIME and CGC only using two-dimensional simulated ground truth. While PMIME appears to be robust across all settings, the application of PMIME in clinical settings may benefit from additional three-dimensional simulation assessment, which may incorporate personalized geometry or cardiac electrophysiology properties.

In applying PMIME to optical mapping data of rat VF, we demonstrated that network measures correlated well with existing organization markers. Unexpectedly, the correlation is inverse to what the nomenclatures imply when applied to undirected unconditioned analysis [31]. Specifically, the coupling of multiple disorganized wavefront events was sufficient to elicit a high network degree and is characterized by higher connectance, larger and fewer clusters. Finally, using a clinical dataset of electrocardiographic imaging in human VF we show that PMIME correlates with DF in determining causality and could be used to identify driver regions that could guide ablation. There are some limitations to our clinical VF data. Firstly, ECGI has not been validated for VF and may not fully capture the complexity of epicardial wavefronts in VF [46]. Additionally, as the subjects were undergoing defibrillation testing, our analysis was limited to the first few seconds of VF. We therefore were unable to describe the pattern of VF in more ischaemic conditions.

Clinical VF has not been extensively studied given the challenges of obtaining data from what is invariably a fatal rhythm if not promptly treated. Haissaguerre *et al.* have recently described non-invasive and intracardiac mapping in 54 patients with clinical VF [3]. They described drivers arising from the Purkinje network and myocardial substrate in early organized VF before degeneration into disorganized VF. Our data support the early period of organized VF in our distinct clinical context. These findings are in broad agreement with previous descriptions of VF intraoperatively during cardiac surgery [1,47–49], and in pre-clinical study [50].

While our analysis focuses on VF data, the signal analysis process is also applicable to AF, where there is more potential for clinical application. The current one-size-fits-all approach in ablation for persistent AF has limited efficacy [7]. Many additional lesion sets have been described and tested; however, although some have shown promise, none have been found to be universally effective [9]. A more personalized approach based on AF mechanisms or spatio-temporal organization is a potential alternative strategy [2]. The challenge with an individualized strategy however is accurate description of AF mechanisms with the limitations of clinical data. We have shown that PMIME-based signal analysis has the potential to overcome some of the limitations of other strategies and may be suitable to guide mechanism-based treatments, which may include ablation of fibrillation drivers in some cases. Further studies are needed to evaluate PMIME in clinical AF data.

## Data Availability

The simulation steps and parameters used are described in detail in the Methods section. Experimental data are available from the Zenodo repository: https://doi.org/10.5281/zenodo.8334660. Informed consent was obtained from each patient to participate in the study approved by the London–Harrow Research Ethics Committee (REC reference: 13/LO/1440; harrow.rec@hra.nhs.uk). Data cannot be shared outside the scope of this strict approval. For any data access, a new application would have to be made to the research ethics committee and have approval of the principal investigator, and would also require new consent from each patient for use of data for other purposes. Implementation of the PMIME analysis is available from the website of its author [29]. Electronic supplementary material is available [51].
